# Pregabalin Attenuates Excitotoxicity in Diabetes

**DOI:** 10.1371/journal.pone.0065154

**Published:** 2013-06-13

**Authors:** Chin-Wei Huang, Ming-Chi Lai, Juei-Tang Cheng, Jing-Jane Tsai, Chao-Ching Huang, Sheng-Nan Wu

**Affiliations:** 1 Department of Neurology, National Cheng Kung University Hospital, College of Medicine, National Cheng Kung University, Tainan, Taiwan; 2 Department of Pediatrics, Chi-Mei Foundation Medical Center, Tainan City, Taiwan; 3 Department of Pharmacology, National Cheng Kung University, Tainan, Taiwan; 4 Department of Pediatrics, National Cheng Kung University Hospital, Tainan, Taiwan; 5 Department of Physiology, National Cheng Kung University, Tainan, Taiwan; Institut National de la Santé et de la Recherche Médicale, France

## Abstract

Diabetes can exacerbate seizures and worsen seizure-related brain damage. In the present study, we aimed to determine whether the standard antiepileptic drug pregabalin (PGB) protects against pilocarpine-induced seizures and excitotoxicity in diabetes. Adult male Sprague-Dawley rats were divided into either a streptozotocin (STZ)-induced diabetes group or a normal saline (NS) group. Both groups were further divided into subgroups that were treated intravenously with either PGB (15 mg/kg) or a vehicle; all groups were treated with subcutaneous pilocarpine (60 mg/kg) to induce seizures. To evaluate spontaneous recurrent seizures (SRS), PGB-pretreated rats were fed rat chow containing oral PGB (450 mg) for 28 consecutive days; vehicle-pretreated rats were fed regular chow. SRS frequency was monitored for 2 weeks from post-status epilepticus day 15. We evaluated both acute neuronal loss and chronic mossy fiber sprouting in the CA3 area. In addition, we performed patch clamp recordings to study evoked excitatory postsynaptic currents (eEPSCs) in hippocampal CA1 neurons for both vehicle-treated rats with SRS. Finally, we used an RNA interference knockdown method for Kir6.2 in a hippocampal cell line to evaluate PGB's effects in the presence of high-dose ATP. We found that compared to vehicle-treated rats, PGB-treated rats showed less severe acute seizure activity, reduced acute neuronal loss, and chronic mossy fiber sprouting. In the vehicle-treated STZ rats, eEPSC amplitude was significantly lower after PGB administration, but glibenclamide reversed this effect. The RNA interference study confirmed that PGB could counteract the ATP-sensitive potassium channel (K_ATP_)-closing effect of high-dose ATP. By opening K_ATP_, PGB protects against neuronal excitotoxicity, and is therefore a potential antiepileptogenic in diabetes. These findings might help develop a clinical algorithm for treating patients with epilepsy and comorbid metabolic disorders.

## Introduction

In modern societies, more than one-third of the adult populations develops metabolic syndrome (MS) [1]. Due to its major role in MS, diabetes and its complications deserve attention [2]. Both clinical and laboratory studies [3]–[5] report that MS and diabetes increase seizure severity, which can significantly affect neurological outcome. We showed previously that diabetes worsened seizure-induced brain damage and impaired both cognition and synaptic plasticity [6].

Using *in vitro* hippocampal neurons, we demonstrated that ATP-sensitive potassium channel (K_ATP_) agonists could hyperpolarize membrane potentials and attenuate action potential firing when extracellular glucose concentrations are high [7]. The K_ATP_ contains pore-forming units with inward rectifying characteristics (Kir6.1 or Kir6.2) and sulfonylurea (SUR) binding sites (SUR1, SUR2A, or SUR2B) [8]. Kir6.2 mRNA is found in cells distributed throughout the brain [9] and overlaps considerably with SUR1 mRNA. These observations indicate the Kir6.2/SUR1 complex is an ideal candidate for studying K_ATP_ function in the brain [10], [11].

We previously [12] found that the use of diazoxide to open K_ATP_ could help reduce seizure severity and protect against seizure-induced hippocampal damage in diabetes. However, alteration of K_ATP_ function in diabetes has been reported [14]. Furthermore, it is uncertain whether the K_ATP_ agonist diazoxide can affect blood glucose levels [13]. In this study, we aimed to determine whether standard antiepileptic drugs (AEDs) could benefit seizure activity in diabetes by opening K_ATP_ *in vivo*.

Pregabalin (PGB, S-enantiomer of 3-aminomethyl-5-methylhexanoic acid), a standard AED, is more effective in controlling seizures [15] than its analogue gabapentin [16]. It is well established that PGB can relieve diabetic neuropathic pain and anxiety disorder [17], [18]. PGB modulates neuronal excitability by binding with the alpha-2-delta subunit of P/Q-type calcium channels [19]. We previously identified a unique mechanism in which PGB opened K_ATP_ in hippocampal neurons *in vitro* by modulating the probability of K_ATP_ opening, but not unitary conductance [20]. Whether this observation is relevant to *in vivo* studies of diabetes, however, remains unknown.

Patients with epilepsy must carefully monitor their blood glucose levels to attenuate seizure severity. Attenuation of seizure severity could subsequently help prevent long-term metabolic and vascular complications resulting from AED therapy [21]. Both traditional and some newer AEDs, including phenytoin, carbamazepine, valproic acid, and topiramate, can affect blood glucose stability [23]–[26]. Unlike other AEDs, PGB shows no obvious effects on blood glucose levels [22]. Thus, we aimed to determine whether PGB has a distinct effect on pilocarpine-induced neuronal excitotoxicity in diabetes.

## Materials and Methods

Experiments were conducted in accordance with the guidelines of Experimental Ethics Committee of National Cheng Kung University. Procedures for animal experimentation were reviewed and approved by the Institutional Animal Care and Use Committee.

### Animals

Adult male Sprague-Dawley rats weighing 180–200 g were purchased from National Cheng Kung University. They were housed in the university's Animal Center and allowed free access to water and a pelleted rodent diet (Richmond Standard; PMI Feeds, St. Louis, MO). Efforts were made to reduce the number of rats used.

### *In Vivo* experiments

#### Grouping

**Figure 1:**
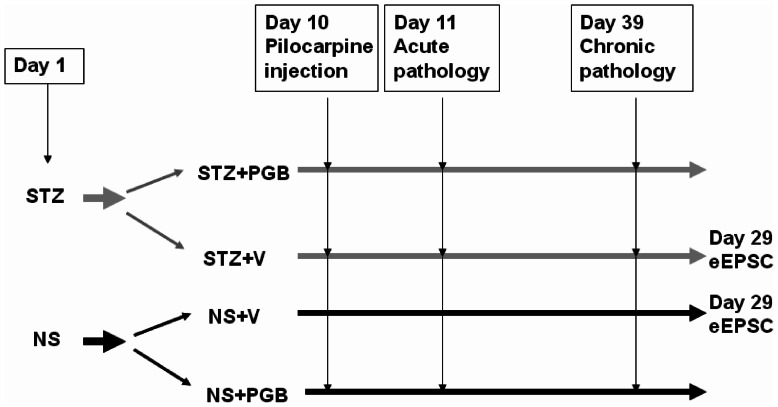
Experimental protocol. **Pilocarpine (60** **mg/kg; s.c.) was administered to induce seizures on day 10 (n = 30 in each group).** Lithium chloride (3 meq/kg; i.p.) and methylscopolamine (25 mg/kg; s.c.) were injected 18–20 h and 30 min before the pilocarpine injection, respectively [12]. Twenty-four hours after pilocarpine-induced status epilepticus (day 11), acute pathological study on neuronal loss was performed (n = 5 in each group). Twenty-eight days after status epilepticus (day 39), chronic pathological study on mossy fiber sprouting was performed (n = 5 in each group).

On day 1, the rats were divided into the following 2 groups: the streptozotocin (STZ) group, which was injected intravenously (i.v.) with 70 mg/kg of STZ to induce diabetes, and a control group injected (i.v.) with normal saline (NS) (Fig. 1). On day 10, status epilepticus was induced in both groups by subcutaneously (s.c.) injecting lithium chloride, and then 18–20 h later by injecting (s.c.) pilocarpine. We injected the rats with methylscopolamine 30 min before the pilocarpine injection to reduce the peripheral consequences of pilocarpine [12]. To evaluate the effect of PGB on pilocarpine-induced acute seizures, we further divided the groups into subgroups that were given either PGB (15 mg/kg; i.v.) (STZ+PGB, NS+PGB) or a vehicle (V) containing NS and dimethyl sulfoxide (DMSO; NS:DMSO, 1∶3; i.v.) in a volume equal to the PGB dose (STZ+V, NS+V). PGB and V injections were given 20 min before the pilocarpine injection. To evaluate the effect of PGB on chronic pilocarpine-induced seizures and excitotoxicity, we gave STZ+PGB and NS+PGB rats PGB (450 mg/day) in their regular daily chow for 28 consecutive days, beginning 24 h after status epilepticus. STZ+V and NS+V rats were given regular chow during this 28-day period.

#### Measuring glucose concentrations

Plasma glucose concentration was measured in all rats on day 10, before status epilepticus was induced. We measured plasma glucose concentration by snipping each rat's tail and collecting a small blood sample (∼5 μL) on a test strip (Optium blood glucose electrode; Abbott Diabetes Care Inc., USA) that was inserted into a glucose meter (MediSense Optium Xceed, Abbott). The glucose level reading was available in 20 s and was measured in milligrams per deciliter (mg/dL). Rats in the STZ group with fasting blood glucose levels lower than 126 mg/dL were excluded from the study [6]. We again measured plasma glucose concentration 15 min after PGB or V injections (before pilocarpine-induced seizures) to determine the effect of PGB treatment on plasma glucose concentration. The final plasma glucose measurement was performed on day 28 to determine the effect of chronic PGB treatment on plasma glucose concentration.

#### Lithium-pilocarpine seizure modeling

On day 10, all rats were injected with lithium chloride (3 meq/kg; i.p.) and methylscopolamine (25 mg/kg; s.c.), before seizures were induced with pilocarpine injections (60 mg/kg; s.c.). All rats demonstrated behavioral characteristics during epileptic seizures that were similar to those reported previously [6], [27], [28]. Mouth and facial movements, head bobbing and nodding, scratching, masticatory automatisms, and exploratory behavior were observed within 15 to 20 min (stages 1–2). Myoclonic movements of the head and forelimbs (stage 3) began within 20 to 25 min, and progressed to status epilepticus (stages 4–5), with rearing and falling, approximately 50 min after pilocarpine injection. Diazepam (5 mg/kg, i.p.) was administered to reduce seizure activity if status epilepticus lasted for 90 min [29]. We determined the seizure onset latency by measuring the time interval between pilocarpine injection and the onset of overt seizure behaviors (stage 3). The rats were monitored for 24 h following status epilepticus, and supportive care was implemented, including body temperature maintenance, feeding, and adequate hydration. Status epilepticus-related mortality was calculated during the first 24 h after onset.

For chronic evaluation, we began monitoring seizure frequency 14 days after status epilepticus. Rats were monitored with a video camera mounted above the cage for 4 h per day, over 14 consecutive days. A trained technician blinded to the experimental design examined the videos for seizure behavior (i.e., running, jumping, rearing, lordosis, and erect tail). When seizure-like activity was observed, the video was reviewed to confirm seizure behaviors.

### Histopathology

#### Acute stage: cresyl violet staining

Twenty-four hours after status epilepticus, the rats were anesthetized with an overdose of pentothal (60 mg/kg; i.p., n = 5 in each group), and their brains were removed and stored at −80°C. Coronal sections (20-μm thick) of the hippocampus were fixed in formaldehyde for cresyl violet staining as previously described [30]. The cresyl violet-stained sections were examined for gross indications of hippocampal damage. Cells were counted in Nissl-stained sections (10-μm thick) that were magnified (×400) using a computerized image analysis system (Image Plus 2.0; Motic, Richmond, British Columbia, Canada). The hippocampal subfields were defined by an imaginary line connecting the tips of the granule cell layer blades, which separated the cornu ammonis (CA) 3c (medially) from CA3b (laterally), and CA2 from CA1 (Huang et al., 2009). The severity of neuronal damage in the different hippocampus subfields was scored semi-quantitatively by using the following scale: 0 =  no damage; 1 =  less than 10% neuron loss; 2 =  between 11% and 50% neuron loss; and 3 =  greater than or equal to 50% neuron loss [31]. An investigator blind to the study design determined these values for the different groups. The mean values for each group were also determined.

#### Chronic stage: Timm's staining

The brains were removed 28 days after status epilepticus, and coronal sections were cut (20-μm thick) through the entire hippocampus on a freezing microtome. Every sixth section was stained using Timm's stain [32]. The sections were developed in the dark for 10–45 min in 200 ml of a solution containing citric acid (5.1 g), sodium citrate (4.7 g), hydroquinone (3.47 g), AgNO_3_ (212.25 mg), and 120 ml of 50% Arabic gum. Timm's staining was assessed from the septal area to the temporal hippocampus (the region extending from 2.8 to 3.8 mm posterior to the bregma). We used a semi-quantitative scale to evaluate the extent of mossy fiber sprouting in the pyramidal and infrapyramidal areas of the hippocampal CA3 region, and the granular cell and inner molecular layers of the dentate gyrus [30]. The scoring criteria were as follows: 0 =  no granules, 1 =  occasional discrete granule bundles, 2 =  occasional-to-moderate granules, 3 =  prominent granules, 4 =  prominent near-continuous granule band, and 5 =  continuous or nearly continuous dense granule band.

### Western blotting of Kir6.2 protein expression

Twenty-eight days after status epilepticus, the brains of the rats with chronic seizures were removed. The hippocampus was dissected and put in an ice-cold homogenized buffer containing 10 mM Tris-HCl (pH 7.4), 20 mM EDTA, 10 mM EGTA, 20 mM b-glycerophosphate, 50 mM NaF, 50 mM sodium pyrophosphate, 1 mM phenylmethylsulfonyl fluoride, and the protease inhibitors leupeptin (25 mg/ml) and aprotinin (25 mg/ml). The mixture was centrifuged at 1,000 *g* for 10 min at 4°C. The supernatant containing the membrane fraction was then centrifuged at 48,000 *g* for 30 min at 4°C. The supernatant was removed, and the pellet was resuspended on ice in Triton X-100 lysis buffer for 30 min, homogenized, and then centrifuged at 14,010 *g* for 20 min at 4°C. Finally, the supernatant was transferred to a new Eppendorf tube and stored at −80°C. The membrane extracts (20–80 mg) were separated using SDS-polyacrylamide gel electrophoresis, and the proteins were transferred onto a polyvinylidene fluoride (PVDF) membrane (BioTrace^TM^; Pall Corporation, Pensacola, FL). After the proteins were blocked, Western blots were developed using antibodies for Kir6.2 (Santa Cruz Biotechnology, Santa Cruz, CA). The blots were subsequently hybridized using horseradish peroxidase-conjugated anti-goat IgG (Jackson ImmunoResearch Laboratories, West Grove, PA), and developed using a reagent (Western Lightning Chemiluminescence Reagent PLUS; PerkinElmer Life Sciences, Boston, MA). The blot was incubated with goat polyclonal antibody (1∶1000) to bind the actin (Santa Cruz Biotechnology, Inc., Santa Cruz, CA, USA). The immunoblots for Kir6.2 and actin were quantified using a laser densitometer.

### *In vitro* experiments

#### Preparing and maintaining hippocampal slices

Twenty-eight days after status epilepticus, the rats in the STZ+V and NS+V groups were deeply anesthetized with ether and immediately decapitated. Their brains were removed and immersed in ice-cold artificial cerebrospinal fluid (ACSF). The ACSF consisted of 124 mM NaCl, 25 mM NaHCO_3_, 3 mM KCl, 1.24 mM KH_2_PO_4_, 1.4 mM MgSO_4_, 2.2 mM CaCl_2_, and 10 mM glucose, and was continuously aerated with 95% O_2_ and 5% CO_2_ (pH 7.4) at 4°C. Transverse hippocampal slices (400-μm thick) were prepared using a vibrating tissue slicer (DTK-100; Dosaka EM, Kyoto, Japan). The slices were then pre-incubated in ACSF for at least 1 h at 30°C. Slices from the STZ rats were maintained in 20 mM glucose. The NaCl concentration in the ACSF was adjusted to maintain an osmolarity of 280–290 mOsm.

#### Slice electrophysiological recording

Slices were transferred to a recording chamber, held in place with a nylon mesh, and perfused continuously (at a rate of 2–3 ml/min) with carbogenated ACSF containing bicuculline methiodide (10 μM) to block GABAA receptor-mediated inhibitory synaptic currents. eEPSCs were recorded at 32°C (pH  = 7.2–7.3) from CA1 pyramidal neurons by using the patch-clamp technique in the whole-cell configuration. Recording pipettes were pulled from borosilicate glass capillaries with an inner filament on a pipette puller (P-2000; Sutter Instrument, Novato, CA). The pipettes (resistance range: 3–6 MΩ) were filled with a solution containing 132.5 mM Cs-gluconate, 17.5 mM CsCl, 2 mM MgCl_2_, 0.5 mM EGTA, 10 mM HEPES, 4 mM ATP, and 5 mM QX-314, with the pH adjusted to 7.2 by using CsOH. Excitatory postsynaptic responses were evoked by stimulating the Schaffer collateral-commissural pathway via a constant current pulse (0.05 ms) delivered through a tungsten bipolar electrode. Whole-cell recordings of CA1 neurons in brain slices were made using the “blind” method with an amplifier (MultiClamp 700A; Molecular Devices, Sunnyvale, CA).

A seal resistance of at least 1 GΩ and access resistance less than 20 MΩ that changed by less than 15% during a recording, were considered acceptable. Series resistance was optimally compensated. eEPSCs were recorded while CA1 neurons were voltage clamped at –60 mV. The eEPSCs were performed within 10 min of establishing the whole-cell configuration, and the holding current achieving a steady state. Recordings were performed using pClamp 9.0 (Molecular Devices). Whole-cell currents were filtered at 2 kHz, digitized at 10 kHz (DigiData 1200 Interface; Molecular Devices), and stored on a personal computer for offline analysis. Input resistance was monitored by frequently applying a 10-mV hyperpolarizing voltage step (100 ms duration) from a holding potential of −60 mV. Data were analyzed using pCLAMP 9.0 and Origin 8.0 (Microcal Software, Northampton, MA).

#### Cell preparation

The H19-7 cell line (CRL-2526; American Type Culture Collection, Manassas, VA) was originally derived from hippocampi dissected from 17-day-old Holtzman rat embryos and immortalized using retroviral transduction of temperature-sensitive tsA58 SV40 large T antigen [7], [20], [33]. The cells were maintained in flasks coated with 15 μg/mL of poly-l-lysine, and in Dulbecco's modified Eagle's medium with 4 mM l-glutamine that was adjusted with sodium bicarbonate (1.5 g/L), glucose (4.5 g/L), G418 (200 μg/mL), and puromycin (1 μg/mL), and supplemented with 10% fetal bovine serum. The cells were equilibrated in a humidified atmosphere of 5% CO_2_/95% O_2_ and a permissive air temperature of 34°C. For the rat Kir6.2 RNAi knockdown study, the cells were plated on 6-well plates. The cells were ready for transfection when they reached 30–50% confluence, which was typically 18–24 h after seeding.

### Small interfering RNA transfection

Custom stealth RNAi for rat Kir6.2 sense: 5′-AUAUUCUGCACGAUCAGAAUAAGGA-3′, antisense: 5′-UCCUUAUUCUGAUCGUGCAGAAUAU-3′ and scrambled negative control siRNA, which does not interfere with any known mRNA, were purchased from Invitrogen (Carlsbad, CA). H19-7 cells were transfected with siRNAs (25 nM) by using a transfection reagent (Lipofectamine RNAiMAX; Invitrogen, Carlsbad, CA) in accordance with the manufacturer's protocol and as previously described [34], [35]. Briefly, gene-specific siRNA oligomers were diluted in a reduced serum medium (Opti-MEM I; Invitrogen, Carlsbad, CA) and mixed with the transfection reagent. The complex was incubated at room temperature for 20 min and then added to the cells. Transfected cells were incubated at 34°C for 48 h before they were harvested. After incubation, the cells were examined with patch-clamp recordings to evaluate channel function.

### Reverse transcriptase-polymerase chain reaction

The RNAi knockdown efficiency of rat Kir6.2 mRNA in the H19-7 cells was determined using a semi-quantitative reverse transcriptase-polymerase chain reaction (RT-PCR). Total RNA was extracted using a reagent (Trizol; Invitrogen, Carlsbad, CA) and reverse transcribed into cDNA by using reverse transcriptase (Superscript III; Invitrogen, Carlsbad, CA). The sequences of Kir6.2 primers used for PCR were [36]: 5′-GCAGAGGACCCTACAGAGCC-3′ and 5′-GCGGCCATGTCGCAGGGTGA-3′.

GADPH was the positive internal control. Rat Kir6.2 and GADPH mRNA were amplified using a reagent (PCR SuperMix; Invitrogen, Carlsbad, CA). PCR products were analyzed on 1.5% (w/v) agarose gel containing ethidium bromide and then visualized under ultraviolet light. Optical densities of DNA bands were scanned and quantified (Scion Image software; Scion, Frederick, MD).

### Cellular electrophysiological recording

Immediately before each experiment, the cells were dissociated and an aliquot of cell suspension was transferred to a recording chamber mounted on the stage of an inverted microscope (DM-IL; Leica Microsystems, Wetzlar, Germany). The microscope was coupled to a digital video camera (DCR-TRV30; Sony, Japan) with a magnification of up to 1500× to monitor cell size during the experiments. The cells were immersed at room temperature (20–25°C) in normal Tyrode's solution. The composition of normal Tyrode's solution was 136.5 mM NaCl, 5.4 mM KCl, 1.8 mM CaCl_2_, 0.53 mM MgCl_2_, 5.5 mM glucose, and 5.5 mM HEPES-NaOH buffer (pH 7.4). For single-channel recordings, the high K^+^ bathing solution contained 145 mM KCl, 0.53 mM MgCl_2_, and 5 mM HEPES-KOH (pH 7.4); the pipette solution contained 145 mM KCl, 2 mM MgCl_2_, and 5 mM HEPES-KOH (pH 7.2). Patch pipettes were pulled from borosilicate glass capillaries, and had resistances ranging between 3 and 6 MΩ when immersed in normal Tyrode's solution. Ion currents were measured in a whole-cell configuration. All potentials were corrected for liquid junction potential.

Single-channel currents from K_ATP_ were analyzed using pCLAMP 9.0. Multi-Gaussian adjustments of the amplitude distributions between channels were used to determine unitary currents. The number of active channels in patch N was counted at the end of each experiment and used to normalize the opening probability at each potential. The opening probabilities were evaluated using an iterative process to minimize the χ^2^ calculated with sufficient independent observations.

### Drugs and solutions

STZ, lithium, scopolamine, glibenclamide, and pilocarpine were purchased from Sigma-Aldrich (St. Louis, MO) and PGB from Pfizer Pharmaceuticals. All other chemicals, unless otherwise noted, were locally purchased and of reagent grade.

### Statistical analysis

Significance was set at p<0.05 for all statistical tests. The Shapiro-Wilk test was used to determine whether the data were normally distributed. Normally distributed continuous variables were assessed using *t*-tests or one-way analysis of variance (ANOVA), and then Fisher's Least Significant Difference tests (SPSS 15.0; SPSS Institute, Chicago, IL). When the data were not normally distributed, analyses were performed with the non-parametric Kruskal-Wallis H test and Dunn's multiple comparison tests. Analyses were performed using χ^2^ tests, the Yates χ^2^ test, or Fisher's exact probability for nominal variables test. Continuous data are expressed as means ± standard error of the mean (SEM), unless otherwise indicated.

## Results

### PGB treatment did not affect blood glucose level in either the STZ or NS group

On day 10, blood glucose levels were significantly higher in STZ rats (352.1±17 mg/dL) than in NS rats (105.0±4 mg/dL, *p*<0.01). In addition, PGB did not affect plasma glucose levels in either PGB group (STZ+PGB  = 357.4±12 mg/dL vs. STZ+V  = 350.7±17 mg/dL; NS+PGB  = 106±7 mg/dL vs. NS+V  = 104±8 mg/dL, all *p*>0.1). Furthermore, chronic PGB treatment did not significantly affect plasma glucose levels. On day 28, the plasma glucose levels in each group were similar between STZ groups (STZ+PGB  = 320.7±8 mg/dL vs. STZ+V  = 321.4±6 mg/dL, all *p*>0.1) and between NS groups (NS+PGB  = 98.8±6 mg/dL vs. NS+V  = 99.4±5 mg/dL, all *p*>0.1).

### PGB-treated rats had fewer severe acute seizures and less status epilepticus-related mortality

**Figure 2:**
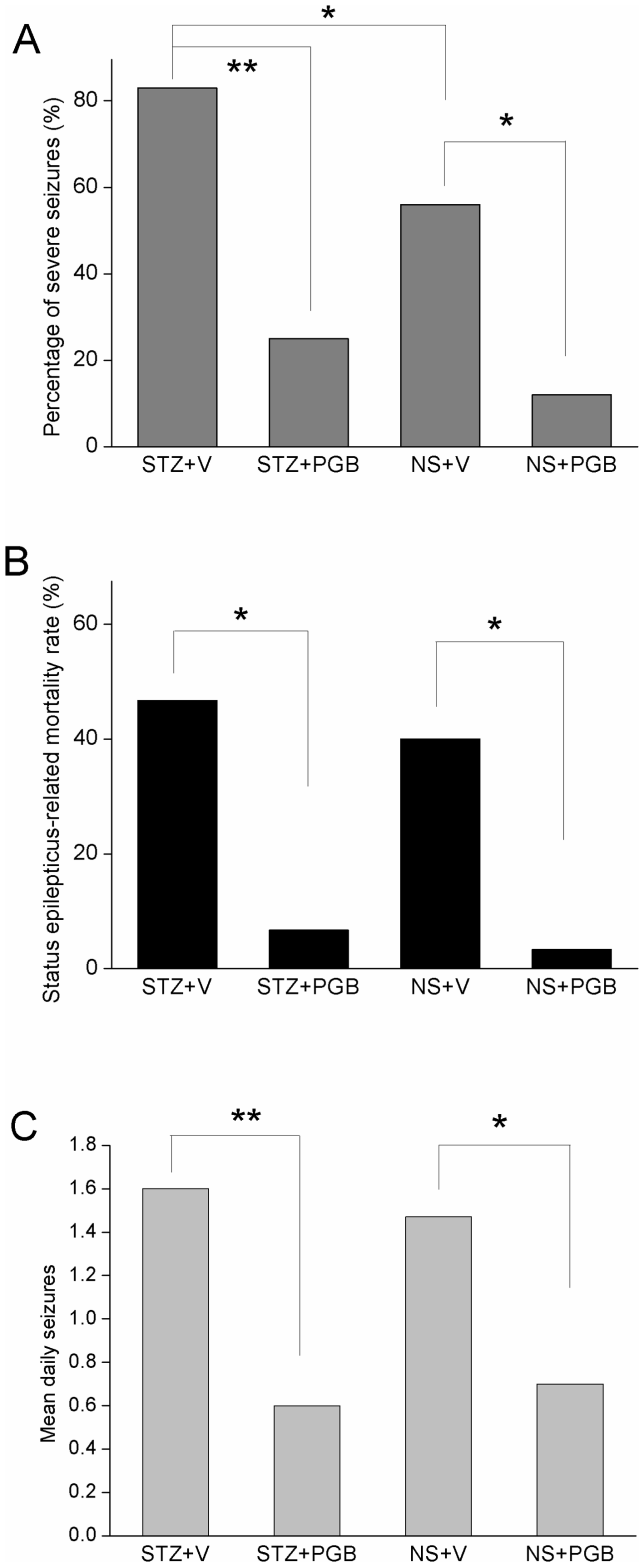
Pilocarpine-induced seizures in the streptozotocin (STZ)-induced diabetes and normal saline (NS) groups. **A:** The STZ+PGB (pregabalin) group had a significantly lower percentage of severe seizures (stages 3–5) than did the STZ+V (vehicle) group (*p<*0.01). Similarly, the NS+PGB group had a lower percentage of severe seizures than the NS+V group did (*p<*0.05). **B:** Status epilepticus-related mortality was significantly higher in the STZ+V group than in the STZ+PGB group (*p<*0.05, n = 30 in each group), and was significantly higher in the NS+V group than in the NS+PGB group (*p<*0.05, n = 30 in each group). **C:** There were significantly fewer spontaneous recurrent seizures (mean daily seizures) in the PGB groups than in the non-PGB groups (STZ+V  = 1.6; STZ+PGB  = 0.6; NS+V  = 1.5; NS+PGB  = 0.7) (χ^2^ test, Fisher's exact test and unpaired *t*-test, n = 10–13 in each group, **p<0.01; *p<0.05).

Compared to the STZ+V group, the STZ+PGB group showed significantly fewer severe acute seizures (stages 3–5) (STZ+PGB  = 26.7% [8/30] vs. STZ+V  = 83% [25/30], *p*<0.01) and lower status epilepticus-related mortality (STZ+PGB  = 6.7% [2/30] vs. STZ+V =  46.7% [14/30], *p*<0.05) (Figs. 2–A, B). Compared to the NS+V group, the NS+PGB group had fewer severe seizures (NS+PGB  = 13% [4/30] vs. NS+V  = 56% [17/30], *p*<0.05), and lower status epilepticus-related mortality (NS+PGB  = 6% (2/30) vs. NS+V  = 40% [12/30], *p<*0.05) (Figs. 2–A, B). These findings suggest that PGB provides protection against seizures in diabetic hyperglycemia.

### PGB-treated rats had fewer spontaneous recurrent seizures

After a latent phase of 2 weeks, most of the rats in STZ+V and NS+V groups had SRS (STZ+V  = 91% [10/11], NS+V  = 85% [11/13]), compared to STZ+PGB (50% [11/22]) and NS+PGB (48% [11/23]). The mean number of daily spontaneous seizures was quantified for each rat based on the daily 4-h monitoring period. The mean number of daily seizures was significantly smaller in the PGB groups than in the non-PGB groups (STZ+V  = 1.6, STZ+PGB  = 0.6, *p*<0.01; NS+V  = 1.5, NS+PGB  = 0.7, *p*<0.05) (Fig. 2C).

### PGB-treated rats demonstrated less acute hippocampal damage after status epilepticus

**Figure 3:**
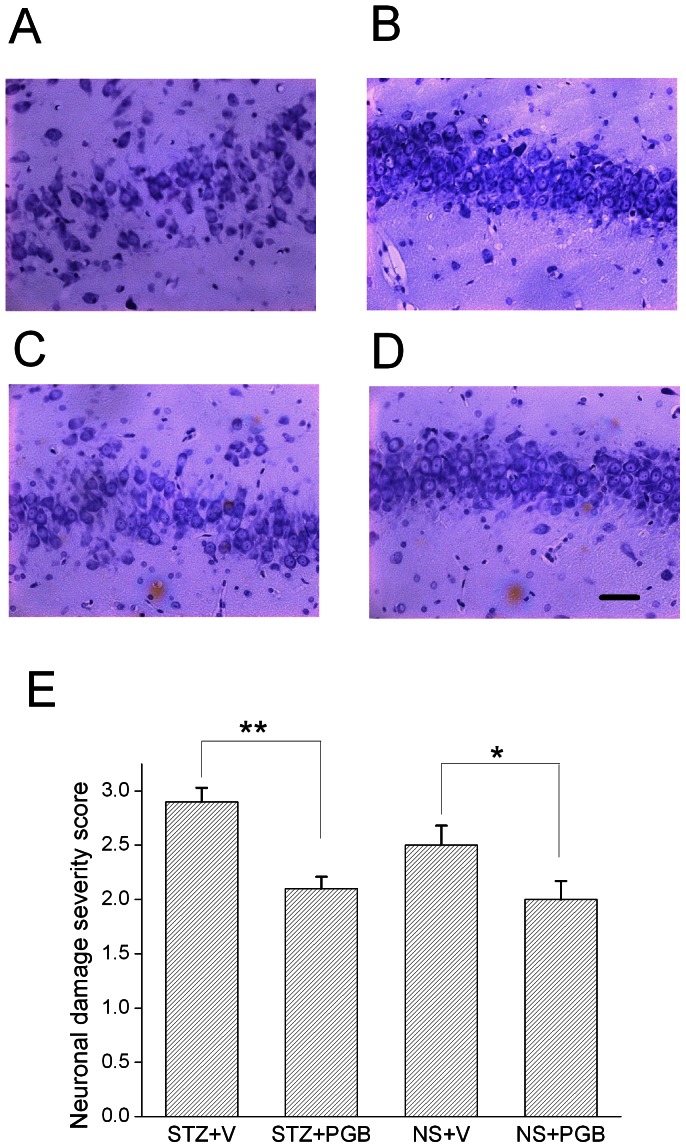
Neuronal loss during the post-status epilepticus acute stage. Twenty-four hours after status epilepticus, cresyl violet staining showed substantially fewer neurons in the hippocampal CA3 area in **A,** the STZ+V group, than in **B,** the STZ+PGB group. There was more neuronal death in the hippocampal CA3 area in **C,** the NS+V group, than in **D,** the NS+PGB group. A semi-quantitative analysis, **E**, showed that the difference between the CA3 areas was significant (STZ+V versus STZ+PGB, *p*<0.01; NS+V versus NS+PGB, *p*<0.05) (Kruskal-Wallis H test and then Dunn's multiple comparison test, n = 5 in each group, **p<0.01; *p<0.05). The scale bar (**D**) represents 40 μM.

Cresyl violet staining revealed fewer neurons in the hippocampal CA3 of the STZ+V rats (Fig. 3A) than in the STZ+PGB rats 24 h following status epilepticus (Fig. 3B). In addition, CA3 neuronal damage was more common in the NS+V rats (Fig. 3C) than in the NS+PGB rats (Figure 3D). A blind semi-quantitative analysis revealed that compared to the STZ+PGB rats, the STZ+V rats had significant hippocampal neuronal damage (STZ+V  = 2.9±0.2; STZ+PGB  = 2.1±0.1, *p*<0.01). The NS+V rats also showed more neuronal damage than the NS+PGB rats (NS+V  = 2.5±0.3; NS+PGB  = 2.0±0.3, *p*<0.05, Fig. 3E). There were no significant differences in cell counts in hippocampal CA1 areas, the hilus, and dentate granular cell layers between the STZ+V and STZ+PGB rats or between the NS+V and NS+PGB rats.

### PGB-treated rats demonstrated less mossy fiber sprouting

**Figure 4:**
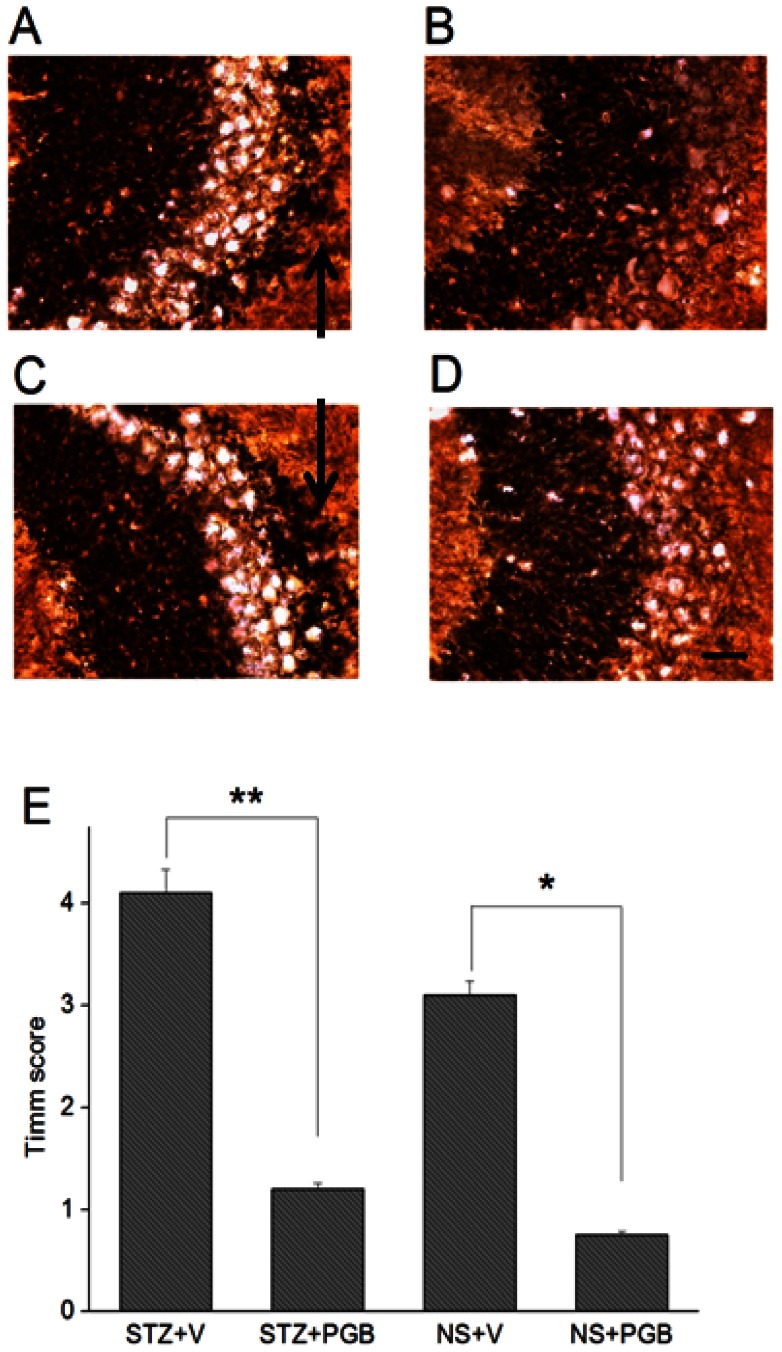
Mossy fiber sprouting in the post-status epilepticus chronic stage. There were more prominently Timm's-stained fibers in the hippocampal CA3 area in **A,** the STZ+V group (strata pyramidale and oriens; *arrow*), than in **B,** the STZ+PGB group. There were also more Timm's-stained fibers in **C,** the NS+V group (strata pyramidale and oriens; *arrow*), than in **D,** the NS+PGB group. **E:** The Timm's score for the quantitative analysis of mossy fiber sprouting in the CA3 region was significantly higher in the STZ+V and NS+V groups than in the other two groups (STZ+V versus STZ+PGB, **p<0.01; NS+V versus NS+PGB, *p<0.05) (ANOVA, n = 5 in each group). The scale bar (**D**) represents 40 μM.

Timm's staining (Figs. 4–A, B, E) revealed at 28 days after status epilepticus, the STZ+V rats showed greater dense mossy fiber sprouting in the hippocampal CA3 region than did the STZ+PGB rats (STZ+V  = 4.1±0.3, STZ+PGB  = 1.2±0.1, *p*<0.01). The NS+V group had a higher Timm's score than the NS+PGB group (NS+V  = 3.1±0.2, NS+PGB  = 0.7±0.1, *p*<0.05) (Figs. 4–C, D, E).

### Kir6.2 expression was decreased in chronically diabetic rats

**Figure 5:**
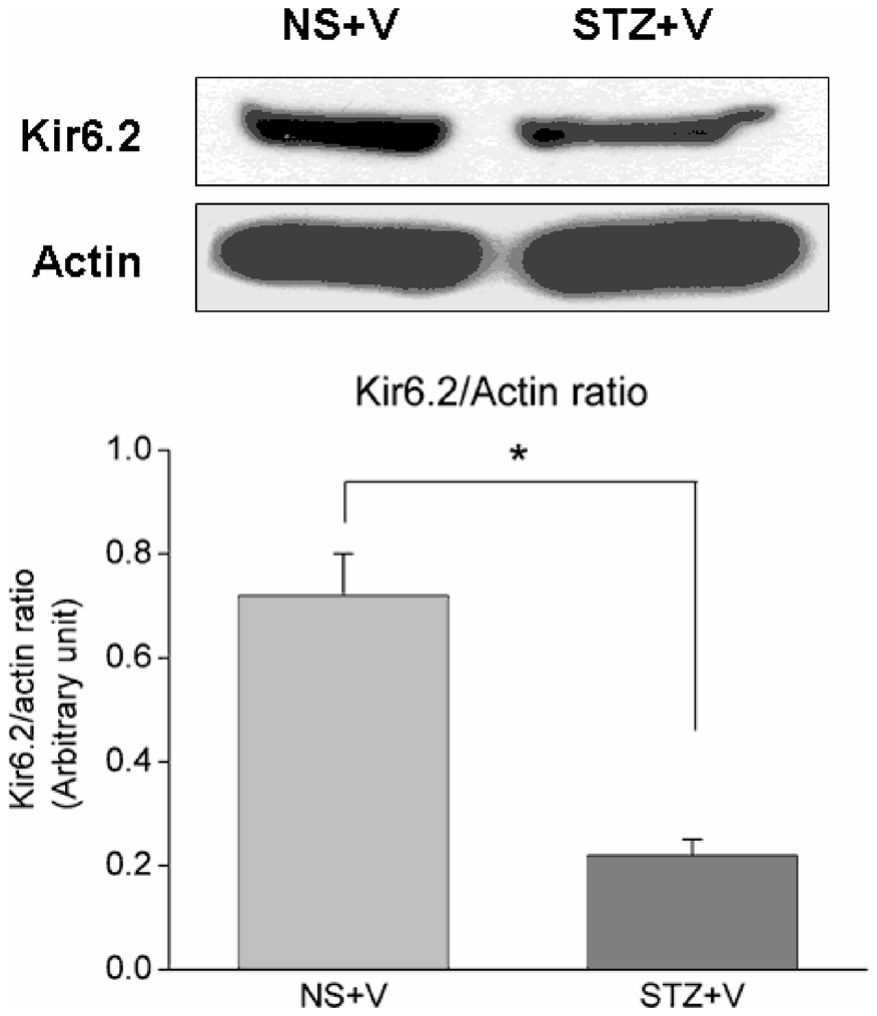
Representative Western blots showing Kir6.2 protein expression in the hippocampus in the NS+V and STZ+V groups. Immunoblots of 100 μg of tissue lysate from rats in the two groups against Kir6.2 antibody. (Unpaired *t*-test, n = 5 per group, *p<0.05).

We investigated Kir6.2 expression in chronically diabetic rats in order to quantify K_ATP_ activity. Western blotting revealed both Kir6.2 and K_ATP_ subunit protein expression in NS+V and STZ+V rats. Quantitative analysis showed that Kir6.2 protein expression was significantly higher in the NS+V group than in the STZ+V group (0.72±0.08 vs. 0.22±0.03, *p<*0.05) (Fig. 5).

### Glibenclamide reversed PGB's effects on CA1-evoked EPSCs in hippocampal slices

**Figure 6:**
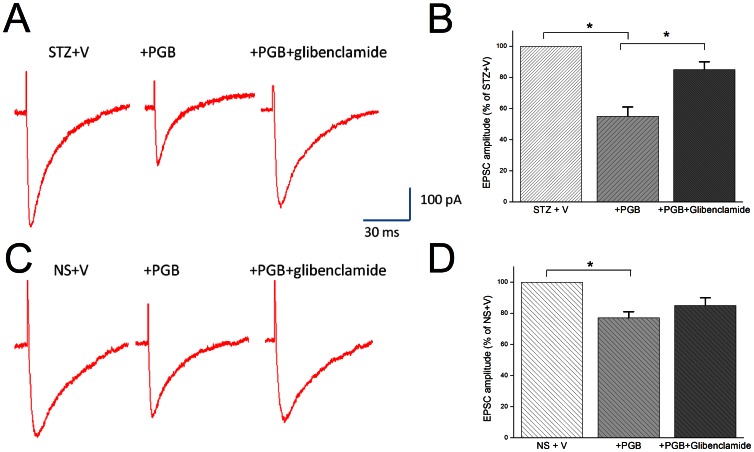
Glibenclamide reversed PGB's effects on the evoked excitatory postsynaptic currents in hippocampal slices. The evoked excitatory postsynaptic synaptic currents (eEPSCs) amplitude in CA1 neurons in the STZ+V and NS+V groups 28 days after status epilepticus. **A, B:** In the STZ+V group, PGB (25 μM) significantly reduced the eEPSC amplitude (55%±6% of baseline amplitude in STZ+V). This reduction was significantly reversed by glibenclamide (10 μM) (85%±6% of baseline amplitude in STZ+V). **C, D:** In contrast, PGB reduced eEPSC amplitude in the NS+V group to a lesser degree (78%±4% of baseline amplitude in NS+V); the reduction was non-significantly reversed by glibenclamide (84%±5% of baseline amplitude in NS+V) (ANOVA, n = 6–7 slices) (*p<0.05).

The CA3 region with its extensive recurrent collateral system, is thought to be a critical site of hippocampal integrity. It's suggested in sharp wave-related population bursts, the coherent discharge of CA3 neurons causes spiking activities in CA1 pyramidal neurons [37]. It's reasonably hypothesized that neuronal excitability at CA1 would be markedly altered in either STZ or NS group in pilocarpine-induced epilepsy, even without marked cell loss. In the STZ+V group, PGB addition significantly reduced eEPSC amplitude (55%±6% of baseline, *p*<0.05), which could be reversed by adding glibenclamide (10 μM) (85%±6% of baseline, *p*<0.05) (Figs. 6–A, B). In the NS+V group, PGB addition significantly reduced the eEPSC amplitude (78%±4% of baseline, *p*<0.05), but glibenclamide could not reverse this effect (84%±5% of baseline) (Figs. 6–C, D). These findings suggest that PGB significantly modulates neuronal excitability in CA1 neurons by opening KATP in diabetic hyperglycemia with pilocarpine-induced epilepsy.

### PGB opened K_ATP_ in the presence of high ATP concentrations in hippocampal neuron-derived H19-7 cells

We used RNAi for Kir6.2 in *in vitro* hippocampal neurons to further examine the protective role of PGB in diabetic hyperglycemia. A hippocampal neuron-derived H19-7 neuronal cell line stably expressing Kir 6.2 and the SUR1 subunit was transfected with siRNAs targeting Kir 6.2. The effects of RNAi were monitored at the mRNA and functional levels. RT-PCR revealed a significant reduction of Kir6.2 mRNA after RNAi (data not shown). The single-channel unitary conductance of the K_ATP_ in the H19-7 neurons was 76±1.8 pS at hyperpolarized potentials, with a reversal potential of 64.6±2.7 mV, which is consistent with inward rectification in K_ATP_.

**Figure 7:**
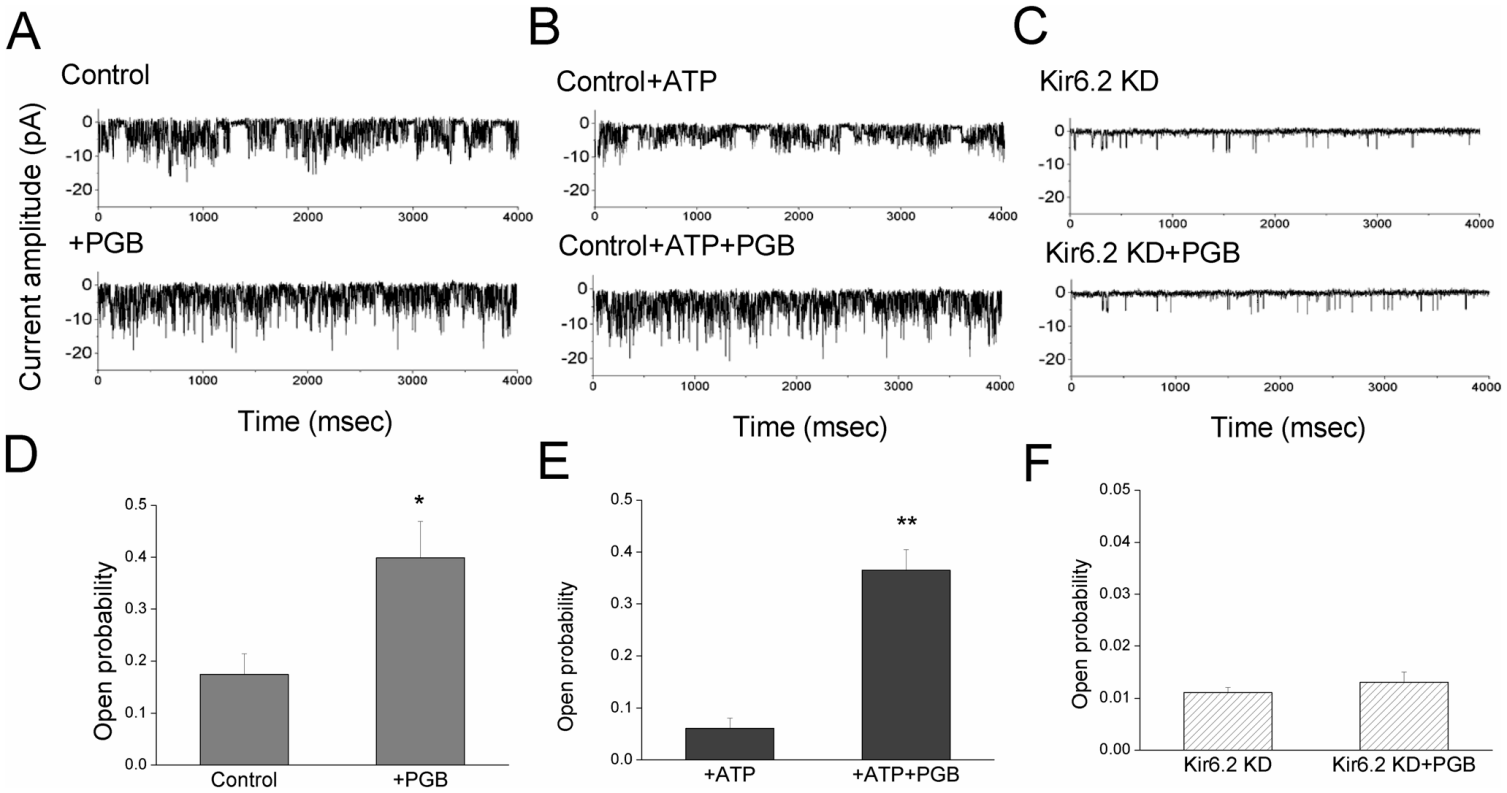
PGB significantly opened K_ATP_ in the presence of a high concentration of ATP. A hippocampal-derived H19-7 neuronal cell line stably expressing Kir6.2 and the SUR1 subunit was transfected with siRNAs targeting Kir6.2. The cells were immersed in a symmetrical K+ (145 mM) solution (holding potential  = −60 mV). Inside-out configuration in patch clamp technology was used. **A, C:** Control neurons showed more channel activities (0.174±0.043) than the RNAi Kir6.2 knockdown (KD) neurons did (0.013±0.002). PGB (50 μM) significantly (*p*<0.05) increased the channel activities to 0.399±0.068 in control neurons, but did not increase the channel activities in the Kir6.2 KD neurons (0.014±0.001). **B:** ATP (1 mM) significantly (*p*<0.05) attenuated the channel activities in control neurons (0.061±0.025). **B, E:** Adding PGB (50 μM) to ATP (1 mM) showed significant increases in channel activity in control neurons (0.365±0.041). **D–F:** In control neurons, the difference in opening probabilities between neurons treated with PGB+ATP and ATP alone was even higher and more significant (*p*<0.01) than that between neurons treated with PGB alone and control (*p*<0.05). **D–F:** Bar graph showing the effect of different treatments on the opening probabilities in **D, E:** control and **F:** Kir6.2 KD neurons. *p<0.05 compared to the control group; **p<0.01 compared to the control+ATP group (ANOVA and Fisher's Least Significant Difference tests, n = 5–8).

Using the inside-out configuration with patch clamp recordings, we found control neurons showed more channel activity (0.174±0.043) than the neurons with RNAi Kir6.2 knockdown (0.013±0.002) (Figs. 7–A, C). The addition of PGB (50 μM) significantly increased channel activity (0.399±0.068) in the control neurons, but not in the neurons with Kir6.2 knockdown (0.014±0.001; Figs. 7–A, C). The presence of ATP (1 mM) significantly attenuated channel activity in the control neurons (0.061±0.025) (Fig. 7B), but addition of PGB (50 μM) to ATP (1 mM) revealed significantly increased channel activity in control neurons (0.365±0.041) (Figs. 7–B, E). In the control neurons, the difference in opening probabilities between neurons treated with PGB+ATP or ATP alone was significantly greater (*p*<0.01) than that observed between the neurons treated with PGB alone and control neurons (*p*<0.05) (Figs. 7D–F).

## Discussion

We examined whether PGB, a standard AED with K_ATP_ agonist properties, protected diabetic rats against pilocarpine-induced seizures and excitotoxicity. We found that PGB (1) reduced acute seizure severity and status epilepticus-related mortality, (2) reduced chronic seizure frequency, (3) ameliorated neuronal damage induced by pilocarpine-induced epilepsy and hyperexcitability (these effects were attenuated by a K_ATP_ antagonist), and (4) positively opened K_ATP_ in the presence of a high concentration of ATP. We showed *in vivo* and *in vitro* evidence that PGB protected diabetic rats against hippocampal damage and seizures in a pilocarpine model, and that K_ATP_ opening effects were the underlying mechanism.

Modulated K_ATP_ may alter seizure thresholds and epileptiform activity, both *in vitro* and *in vivo* [38]–[40]. K_ATP_ activation leads to membrane potential hyperpolarization, which further protects neurons by reducing excitotoxicity in pilocarpine-induced status epilepticus [41] or hypoxia [42]. In this study, we showed that, hippocampal Kir6.2 expression is reduced in diabetic rats. This reduction could potentially lead to increased neuronal excitability. Interestingly, in spite of the reduced protein expression, PGB may activate the remaining Kir6.2 and induce neuroprotection. We showed previously [6] that diabetic rats in a pilocarpine-induced seizure model had more severe seizures and neuronal loss than controls, which further indicates diabetic (glucose) neuronal hyperexcitability. We thus hypothesized that, for seizures in patients with diabetes, targeting K_ATP_ will have some benefits in addition to those provided by other AEDs that do not open K_ATP_.

Diazoxide, a well-known K_ATP_ agonist, reduces glutamate release by opening presynaptic K_ATP_ Kir6.2/SUR1 channels [43] and protects diabetic rats from status epilepticus-induced neuronal damage [12]. However, its effect on rats without diabetes was not significant. Compared with diazoxide, PGB had a protective effect, both in control rats and in diabetic rats, but with a more significant effect on the latter. The prominent K_ATP_ modulating effects, in addition to PGB's calcium channel-modulating effects [44], suggest a possible synergistic interaction in treating neuronal hyperexcitability disorder, especially in metabolic disorders that disrupt K_ATP_ activity. The mechanisms underlying excitotoxicity and seizure attenuation could differentiate PGB from diazoxide, and may explain the significant neuroprotective effects in the STZ+PGB group. Even so, the STZ+V group showed higher excitotoxicity than the NS+V group.

Previous reports [45] suggest that diazoxide may transiently elevate blood glucose levels, which can vary among individuals. We found no significant increment of blood glucose levels in control or diabetic rats after a single injection of diazoxide [12]. In the current study, neither acute nor chronic administration of PGB significantly altered blood glucose levels.

Our single-channel study that indicates PGB might involve an intracellular action. It has been proposed that PGB might involve both intracellular and extracellular sites of action, and a prolonged exposure to PGB produces delayed allosteric enhancement of voltage-activated potassium currents, which involves cAMP-dependent protein kinase A-mediated phosphorylation [46]. PGB may be transported into cells via L alpha-amino acid transporters and bind to intracellular sites to open K_ATP_. It is proposed that PGB has an indirect effect on GABA transporter 1, but does not bind to GABA receptors [47]. In addition, it has been proposed that PGB increases glutamate transporter type 3 activity through protein kinase C and phosphatidylinositol-3-kinase [48]. Although the exact binding target site of PGB is currently unknown, our results might suggest PGB's enhanced K_ATP_ opening effect in diabetes is probably related to allosteric modulation or altered K_ATP_ kinetics [20]. The exact intracellular site of action for PGB requires further investigation.

PGB delays epileptogenesis in animal models of pilocarpine-induced epilepsy by protecting the entorhinal and piriform cortices [49]. In the present study, we observed decreased axonal sprouting and fewer seizures in the groups treated with PGB, which supports this notion. Recurrent excitation and the development of seizures have been associated with aberrant mossy fiber sprouting in the hippocampus [50]. Of interest, Ingram et al. [51] reported that inhibition of calcineurin or L-type calcium channels does not block mossy fiber sprouting in the model of pilocarpine-induced epilepsy, which suggests that the standard mechanism used by PGB to modulate high voltage-activated calcium channels has a limited effect on abnormal axonal sprouting. Furthermore, it is known that increased excitability in CA3 pyramidal neurons increases the probability of burst activity and leads to abnormal CA1 synchronization [52]. Although the histopathological findings showed marked cell loss at CA3 in the present study, the eEPSC evaluation at CA1 suggested that functionally altered excitability involves both the CA3-CA1 circuit and PGB's integral effects on the CA3-CA1 network.

It is also interesting to know that PGB is the mainstay treatment for diabetic neuropathic pain. In neuropathic pain, an abnormal neuronal sprouting “wind-up” underlies the increased responsiveness to noxious stimuli and central sensitization. Zoga et al. [53] and Sarantopoulos et al. [54] described the down-regulation of K_ATP_ in peripheral nerve injury, and Santos et al. [55] reported involvement of glibenclamide-sensitive K_ATP_ in hyperalgesia and neuropathic pain. Given this understanding of the shared mechanism between epileptogenesis and neuropathic pain, as well as the specific role of K_ATP_ in diabetes, the direct effects of K_ATP_ opening by PGB on chronic axonal sprouting and epileptogenesis necessitate further investigations.

In addition to epilepsy and neuropathic pain, Yoon et al. [56] showed that PGB improved ischemic stroke outcome by suppressing calcium-mediated proteolysis in a mouse model. Whether this protective effect is partially related to K_ATP_ opening, as was demonstrated previously in a hypoxia model [39], is worth attention. In an animal model of Alzheimer's disease [57], long-term diazoxide treatment improved performance in a learning and memory test and reduced anxiety levels. Our study supports K_ATP_'s role in treating the hyperexcitability disorders, especially in diabetic hyperglycemia.

In conclusion, our study indicates that in diabetic hyperglycemia with seizures, PGB protects against seizures and neuronal excitotoxicity and is potentially antiepileptogenic. This study contributes to our understanding of a clinically applicable algorithm for treating patients with epilepsy and comorbid metabolic disorders.
